# Germline Genetics of Prostate Cancer: Prevalence of Risk Variants and Clinical Implications for Disease Management

**DOI:** 10.3390/cancers13092154

**Published:** 2021-04-29

**Authors:** David K. Doan, Keith T. Schmidt, Cindy H. Chau, William D. Figg

**Affiliations:** 1 Emory University Hospital, Atlanta, GA 30322, USA; doandk@mymail.vcu.edu; 2 Clinical Pharmacology Program, Office of the Clinical Director, Center for Cancer Research, National Cancer Institute, NIH, Bethesda, MD 20892, USA; keith.schmidt@nih.gov; 3 Genitourinary Malignancies Branch, Center for Cancer Research, National Cancer Institute, NIH, Bethesda, MD 20892, USA; chauc@mail.nih.gov

**Keywords:** prostate cancer, genetics, germline testing

## Abstract

**Simple Summary:**

Advances in our understanding of the molecular basis of prostate cancer have resulted in the discovery of a subset of patients harboring germline variants that places them at increased risk of developing the disease. The goal of precision oncology in prostate cancer is to individualize treatments by tailoring them to the genetic characteristics of each patient’s cancer. Management of advanced prostate cancer is rapidly evolving with genomic-driven therapies. We provide a comprehensive overview of the current guideline recommendations for germline testing in prostate cancer. Expanding the use of genetic testing in prostate cancer patients can inform treatment strategies. We discuss prostate cancer germline genomic profiling and its impact on decision making of therapeutic options.

**Abstract:**

Prostate cancer has entered into the era of precision medicine with the recent approvals of targeted therapeutics (olaparib and rucaparib). The presence of germline mutations has important hereditary cancer implications for patients with prostate cancer, and germline testing is increasingly important in cancer screening, risk assessment, and the overall treatment and management of the disease. In this review, we discuss germline variants associated with inherited predisposition, prostate cancer risk and outcomes. We review recommendations for germline testing, available testing platforms, genetic counseling as well as discuss the therapeutic implications of germline variants relevant to prostate cancer treatments. Understanding the role of germline (heritable) mutations that affect prostate cancer biology and risk as well as the subsequent effect of these alterations on potential therapies is critical as the treatment paradigm shifts towards precision medicine. Furthermore, enhancing patient education tactics and healthcare system infrastructure is essential for the utilization of relevant predictive biomarkers and the improvement of clinical outcomes of patients with prostate cancer or at high risk of developing the disease.

## 1. Introduction

Prostate cancer (PCa) is the second most common malignancy worldwide. With an estimated 1.4 million new PCa cases and 375,000 deaths worldwide, the incidence of PCa and mortality rates are expected to continue to increase in the future [1]. In the United States alone, it is estimated that 248,000 men will be diagnosed with PCa, and 34,000 men will die from the disease in 2021 [2]. Compared to other ethnic and racial groups around the world, African American (AA) and Black men have the highest incidence of PCa, mortality rates, and are more likely to develop the malignancy at an earlier age. Risk factors for prostate cancer include age, ancestry, family cancer history, and genetics. Research has shown that socioeconomic status, environmental factors, access to healthcare, and genetics all play a role in the disease course and outcomes [3].

Molecular profiling approaches have sought to tailor oncology treatment to improve patient specific outcomes. The implementation of tumor genomic profiling for the detection of clinically relevant germline variants have enabled significant advances in the treatment of cancers with an inherited component such as breast, ovarian, and colon cancers. Despite such advances in a precision medicine approach for other cancers, PCa treatment options have lagged behind this transition to broader biomarker targeted approaches beyond androgen receptor (AR) pathway modulating therapeutics. Nevertheless, PCa patients have consistently had better outcomes overall with a 5-year relative survival rate of 98.4% compared to lung and breast cancers [4]. Despite these survival rates, treatment resistance and metastatic disease are still commonplace, affecting patient quality of life and outcomes [3]. Androgen-deprivation therapy (ADT) with surgical or medical castration has been the mainstay of treatment for PCa. For advanced prostate cancer and metastatic castration-resistant prostate cancer (mCRPC), the treatment armamentarium includes androgen biosynthesis (e.g., abiraterone) and androgen receptor signaling inhibitors (e.g., enzalutamide), taxane-based chemotherapies (e.g., docetaxel and cabaxitaxel) and immunotherapies such as Sipuleucel-T and pembrolizumab [5]. The recent approvals of rucaparib and olaparib, two poly-ADP ribose polymerase (PARP) inhibitors, have broadened mCRPC treatment, marking perhaps the beginning of expanded approaches in precision medicine using genetics and biomarkers to guide treatment. A genetic biomarker (or genetic marker) is a DNA sequence that causes disease or is associated with disease susceptibility. Variability in the genetic marker affecting expression, function or regulation of the gene can impact therapeutic response. It is of paramount importance to understand the role of germline (heritable) mutations that affect PCa biology and risk as well as the subsequent effect of these alterations on potential therapies.

## 2. Germline Genetic Variations Associated with Prostate Cancer Risk

Evidence that genetics contribute to the risk for prostate cancer stem from genome-wide association studies (GWAS), family-based genetic linkage analyses, and PCa with a family history of other cancers that have inherited mutations in known cancer predisposition genes (e.g., the breast cancer susceptibility genes, *BRCA1* and *BRCA2*). Germline pathogenic variants (PVs) in prostate cancer are rare deleterious alterations that are associated with the development of the disease. PVs in moderate to high penetrant genes are associated with a high lifetime risk of cancer, while common single nucleotide variants (SNVs) identified from GWAS have low to modest effect sizes. SNVs can occur within the regulatory regions of a gene or in the intra- or intergenic regions of DNA. Both types of genetic variants can affect the function of a gene.

Hereditary prostate cancer (HPC) accounts for about 5% to 10% of newly diagnosed PCa cases [6]. So far, known genetic determinants of HPC are deleterious mutations in pan-cancer DNA repair genes (e.g., *BRCA1, BRCA2, ATM*, etc.) or in the PCa-specific risk genes, *HOXB13* [7,8]. Apart from these moderate-to-high risk genes, GWAS using panels of genome-spanning SNVs have identified ~269 low-risk variants associated with prostate cancer incidence, including multiple loci in the 8q24 region, 17q region, and other chromosomes [8,9,10,11]. Polygenic risk scores based on inherited SNVs have been developed for risk stratification and screening [12,13,14,15]; however, their clinical utility for risk assessment, or guiding screening or treatment recommendations remains to be investigated in ongoing studies.

### 2.1. DNA Repair Genes

DNA damage repair (DDR) is a fundamental pathway that ensures the viability of normal and cancerous prostate cells. Mechanisms of DNA repair include base excision repair, nucleotide excision repair, homologous recombination, non-homologous end-joining, direct reversal, mismatch repair, ligation, single-strand break repair, and inter-strand crosslinks [16]. Alterations in any of these mechanisms can increase the risk of developing cancer. Germline variants in DDR genes (e.g., the double-strand break genes, *BRCA1, BRCA2, ATM, ATR, NBN*, *CHEK2, PALB2*, and *RAD51D*, and the mismatch repair genes *MLH1, MSH2, MSH6,* and *PMS2*) are associated with an increased risk for PCa (early onset, aggressive or metastatic disease) [17,18,19,20]. Pathogenic variants in *BRCA2* and *HOXB13* are associated with the highest risk [7,17,21]. Two landmark sequencing studies of germline DNA, Robinson et al. [19] and Pritchard et al. [17], were among the first studies to delineate the prevalence of inherited mutations in DNA repair genes in patients with metastatic prostate cancer. These studies, and others that followed, set the stage for germline genetic testing for prostate cancer to potentially identify genetic risk factors for lethal PCa, risk stratify aggressive disease from indolent PCa, and identify actionable mutations for treatment management. Clinically actionable germline PVs were observed in 8% of mCRPC with 5.3% of individuals harboring *BRCA2* mutations [19].

Men who are carriers of *BRCA1* or *BRCA2* germline mutations have an increased risk for PCa compared to noncarriers and are associated with more aggressive disease [22]. Individuals who are *BRCA2* mutation carriers have a 3-fold elevated risk for high-grade prostate cancer [23]. Pritchard et al. reported an incidence of inherited DDR PVs in patients with metastatic PCa of 11.8% (5.4% with mutations in *BRCA2*, 0.9% in *BRCA1,* 1.9% in *CHEK2*, and 1.6% in *ATM)* [17]. This prevalence was significantly higher compared with patients with localized prostate cancer (11.8% vs. 4.6%) and similar in unselected patients with recurrent or metastatic PCa (6.0% in *BRCA2*, 2.0% in *CHEK2*, and 2.0% in *ATM*) [24]. In a large prospective cohort study, *BRCA2* carriers were found to be at two to five times higher risk of PCa compared to men in the general population [25], consistent with retrospective studies. *BRCA1* carriers are associated with a ~3.75-fold higher relative risk of PCa [26]. Additionally, germline mutations in *BRCA1* or *BRCA2* (and *ATM*) are significantly associated with increased risk for lethal vs. indolent PCa (6.07% vs. 1.44%, respectively) and with earlier age at death and shorter survival time [27]. In Castro et al., *BRCA1*/*BRCA2* mutations were associated with a more aggressive PCa phenotype (Gleason ≥ 8) with a higher probability of nodal involvement and distant metastasis. Subgroup analyses confirmed patients with *BRCA2* PVs were more likely to be associated with poor outcomes [28]. Moreover, germline *BRCA* carriers had worse outcomes than noncarriers when conventionally treated with surgery or radiation therapy for local/locally advanced PCa [29]. Additional studies found that *BRCA2* germline carriers were strongly associated with rapidly progressing lethal prostate cancer [30] and poor survival [31,32]. In a study focusing in tumor profiling of carriers of *BRCA* PVs, higher disease aggressiveness was associated with increased genomic instability and a mutational profile involving genomic/epigenomic dysregulation that more closely resembles metastatic PCa than localized disease [33]. In response to DNA damage, *ATM* is involved in mediating cell cycle arrest, DNA repair, and apoptosis. Carriers of pathogenic *ATM* variants have a four-fold risk of developing PCa and are at an increased risk for early onset disease [34]. Other relevant germline variants in the DDR pathway for metastatic prostate cancer as reported in the literature are listed in Table 1.

### 2.2. Mismatch Repair Genes

Another mechanism to repair DNA damage is a critical pathway known as DNA mismatch repair (MMR). This pathway is another marker associated with increased risk for PCa. Deficiencies in mismatch repair (dMMR), frequently referred to as Lynch Syndrome, can cause microsatellite instability and DNA tandem repeats increasing the rates of replication errors. Lynch syndrome is an autosomal dominant disorder caused by a germline mutation in one of several MMR genes: *MLH1*, *MSH*, *MSH6*, and *PMS2*. Tumors with dMMR have a 1000-fold increase in the frequency of mutations compared to an intact MMR pathway [37]. A systematic review and meta-analysis examined six molecular studies and 18 risk-based studies (that investigated risk of prostate cancer) regarding MMR gene mutations that found that 73% of PCa in mutation carriers were MMR deficient, estimating that carriers have a 3.67-fold increased risk of PCa [38]. Pathogenic *MSH2* variants have a higher risk of prostate cancer with increasing age (23.8% cumulative incidence of PCa by age 75 years versus 13.8% for *MLH1*, 8.9% for *MSH6*, and 4.6% for *PMS2*) [39]. These studies suggest that PCa risk should be considered for inclusion with cancers typically associate with Lynch syndrome spectrum (e.g., colon, ovarian cancer). Thus, dMMR increases the risk of developing PCa with associated mutations causing adverse clinical consequences when they are mutated [40,41]. While deficiencies in MMR-related PCas are rare, accounting for less than 5% of all PCas [19], their detection has therapeutic implications for predicting response to immune checkpoint blockade.

### 2.3. HOXB13

HOXB13, a member of the homeobox (HOX) gene family, is a homeodomain transcription factor that is involved in prostate cancer development. The G84E variant of *HOXB13* was identified by sequencing the 17q21–22 region in four families with pedigrees strongly indicative of hereditary prostate cancer predisposition [7]. G84E was associated with a significantly increased risk (20-fold) of hereditary prostate cancer (PCa case rate 1.4% vs. controls 0.1%) and was more common in men with early onset, familial PCa (3.1%) vs. late-onset, nonfamilial PCa (0.6%) [7]. In a meta-analysis of 25 epidemiological studies (*n* = 145,257 participants; 51,390 cases and 93,867 controls), there was a significant association between G84E and prostate cancer risk (OR 3.248, 95% CI 2.313–4.560) [42]. While this variant has no prognostic implications or utility in distinguishing indolent vs. aggressive disease, G84E could be used for stratifying screening to identify men at high risk [43]. The association between G84E and risk for prostate cancer in mutation carriers (OR 4.81, 95% CI 4.06–5.68) was further confirmed in a separate population-based analysis of approximately 500,000 individuals derived from the UK Biobank (https://www.ukbiobank.ac.uk accessed on 1 March 2021) [44].

### 2.4. Health Disparities in Genetic Studies

The prevalence of these biomarkers and gene mutations among race and ethnicity have not been studied adequately, with limited data in patient populations other than Caucasians. In particular, few datasets on germline DDR mutations in AA men are available owing to low representation in clinical trials and genetic studies, with few studies specifically investigating DDR mutations in the AA population to date [45,46]. Petrovics et al. reported that some germline variants in *BRCA1/BRCA2* are more frequent in AA than white PCa patients (4.6 vs. 1.6%, respectively) [45].

## 3. Recommendation for Germline Genetic Testing in Prostate Cancer

With increased recognition of pathogenic germline alterations in prostate cancer, clinical guidance on the management of these patients is continuously evolving. The National Comprehensive Cancer Network (NCCN, version 2.2021, https://www.nccn.org/professionals/physician_gls/pdf/prostate.pdf, accessed on 1 March 2021) Prostate Cancer Early Detection and Prostate Cancer guidelines recommend genetic testing to patients with a history of high- or very high-risk regional or metastatic prostate cancer, or localized disease with intraductal histology, a family history of high-risk germline mutations (e.g., *BRCA1* and *BRCA2*, Ashkenazi Jewish ancestry), or a strong family history of cancer [47]. Positive findings of germline mutations warrant referral to a cancer genetics expert for further evaluation and management of the disease. In the setting of very-low, low, and intermediate risk disease as well as the absence of a family history of germline mutations, genetic testing would likely yield low detection rates. Studies to evaluate the feasibility of earlier assessment of these markers and the indication for intervention is currently ongoing (e.g., Clinicaltrials.gov Identifier, NCT03805919).

The implementation of germline testing in the setting of PCa was discussed in detail at the Philadelphia Prostate Cancer Consensus in 2019, with the overall recommendations published in March of 2020 [48]. The Consensus determined germline testing to be critical in the hereditary assessment, early detection, treatment, and management of PCa. Genetic testing should be performed in patients with metastatic PCa (castration resistant or sensitive), and men with one brother, father, or two or more male relatives who were either diagnosed with PCa prior to 60 years of age, experienced metastatic disease or died of PCa. Additionally, genetic testing should be considered in patients with nonmetastatic PCa who have Ashkenazi Jewish ancestry, advanced disease (defined as Stage T3a or higher), intraductal or ductal pathology, Grade Group 4 PCa or above, or a family history of two or more relatives on the same side of the family diagnosed with hereditary breast or ovarian cancer, or Lynch Syndrome. A comprehensive genetic panel, including germline mutations of *BRCA1, BRCA2,* MMR genes, and other cancer susceptible genes based on family history, should be used to determine appropriate therapy and clinical trial eligibility. Next-generation sequencing of a tissue biopsy, circulating tumor cells, or cell-free-DNA should be co-implemented to confirm the germline nature of alterations. In the setting of nonmetastatic PCa, reflex testing should be considered, with emphasis on *BRCA2, ATM,* and additional genes based on family and personal history. Lastly, in men who meet the family history criteria but do not have an active PCa diagnosis, reflex testing should be considered to evaluate germline mutations in *BRCA2, HOXB13, BRCA1, ATM*, MMR genes, and other genes related to family or personal history. Reflex testing is particularly important in patients without PCa and patients undergoing active surveillance, as such results allow clinicians to optimally manage the malignancy earlier on in the disease course [48].

Additional guidelines for germline testing are also put forth by other organizations (e.g., 2020 AUA/ASTRO/SUO) and working groups such as the Advanced Prostate Cancer Consensus Conference 2019 consensus recommendations and the Germline Genetics Working Group of the Prostate Cancer Clinical Trials Consortium, both offering clear recommendations for germline testing in patients with PCa [49,50]. A comprehensive summary and comparison of all guidelines were recently conducted by Loeb and Giri [51]. Clinical application of germline testing remains focused on identifying the target population for testing and while most guidelines recommend testing for men with metastatic disease, recommendations for testing in early stage, localized disease are variable.

### Hereditary Genetic Testing Panels

Numerous tests have been developed for comprehensive analysis of DDR and MMR germline mutations. Panels developed by Invitae, Fulgent, Ambry Genetics, GeneDx, and Prevention Genetics, are designed for the evaluation of hereditary prostate cancer risk [52,53,54,55,56]. Additional panels, including the Myriad Genetics myRisk panel and the Color Hereditary cancer panel, expand beyond germline DDR mutations while incorporating other potential genes of interest, including *PTEN, SMAD4,* and *STK11* [57,58]. Genetic testing can be performed on blood or saliva using various platforms that incorporate a panel of defined gene subsets. These panels are summarized in Table 2.

## 4. Germline Markers Relevant for Prostate Cancer Specific Therapeutic Recommendations

In addition to the assessment of prostate cancer risk, germline genetic biomarkers have become relevant in the context of treatment selection in advanced prostate cancer. In 2020, the US FDA approved two PARP inhibitors, olaparib and rucaparib, for the treatment of mCRPC following prior lines of therapy and predictive germline biomarkers, the first such approval for precision medicine guided treatment in prostate cancer. The TOPARP-A study first identified germline DDR mutations associated with favorable outcomes to olaparib therapy in patients with mCRPC previously treated with chemotherapy. Sixteen of 49 evaluable patients achieved therapeutic response (defined by either RECIST v1.1, reduction in PSA of greater than 50%, or CTC conversion) following treatment with olaparib, of which 15 had alterations in DDR genes; 5 of these alterations were germline events in either *BRCA2* or *ATM*. Overall survival was improved in patients with biomarker-positive disease compared to biomarker-negative patients (13.8 vs. 7.5 month; respectively) [59].

The TOPARP-B and PROfound studies were initiated to further validate efficacy of olaparib in patients with DDR mutation-positive mCRPC. TOPARP-B, which implemented a similar composite primary endpoint to TOPARP-A, reported a 46.7% (43 of 92 patients; 95% CI 36.3 to 57.4%) response rate overall. When stratified for specific mutations, patients with *BRCA1/BRCA2* mutations reported the highest response rate of 83.3% (25 of 30 patients; 95% CI 65.3 to 94.4%), with 14 of those patients having a germline mutation. Additionally, a notable response rate was found in patients with mutations in *ATM* (36.8% (7 of 19 patients); 95% CI 16.3 to 61.6%) and PALB2 (57.1% (4 of 7 patients); 95% CI 18.4 to 90.1%), of which germline alterations were present in the 5 patients in the *ATM* sub-cohort and 6 patients in the PALB2 sub-cohort [60].

In contrast to TOPARP-B, PROfound was a randomized, open-label, phase 3 trial designed to detect a difference in imaging-based progression-free survival following treatment with olaparib in patients with mCRPC who were previously treated with abiraterone or enzalutamide and have either a germline or somatic mutations in *BRCA1*, *BRCA2* or *ATM* (Cohort A, *n* = 162). PROfound also enrolled another cohort of patients with other relevant DDR mutations (Cohort B, *n* = 94; e.g., *PALB2, CHEK1/2, BRIP1, CDK12*). In comparison to control, olaparib improved imaging-based progression free survival in Cohort A (7.4 vs. 3.6 month; HR 0.34; 95% CI 0.25–0.47) and in the combined Cohort A and B (5.8 vs. 3.5 month; HR 0.49; 95% CI 0.38–0.63) [61]. The interim overall survival analysis found a significant improvement with olaparib treatment in comparison to control (18.5 vs. 15.1 months; HR, 0.64; 95% CI 0.43–0.97) [61]. Data from the PROfound study led to the approval of olaparib in patients with DDR mutated mCRPC previously treated with enzalutamide or abiraterone. Predictive biomarkers, germline or somatic, for olaparib include *BRCA1, BRCA2, ATM, BARD1, BRIP1, CDK12, CHEK1/2, FANCL, PALB2, RAD51B/C/D,* and *RAD54L*.

In addition to olaparib, rucaparib was also found to provide benefit in patients with DDR mutation positive mCRPC. Rucaparib received FDA approval in *BRCA1/BRCA2* positive (germline or somatic) mCRPC patients previously treated with anti-hormonal and taxane therapy following a 43.5% overall response rate observed on the TRITON2 phase 2 study (27 of 62 patients; 95% CI 31.0–56.7%) [62]. TRITON2 also found limited responses in patients with *ATM* (2 of 19 patients, 10.5%), and *CHEK2* (1 of 9 patients, 11.1%). Among the 14 patients with other alterations, responses were reported in four patients, with mutations in *PALB2, FANCA, BRIP1*, and *RAD51B* [63]. Though not approved by the FDA yet, niraparib was granted “breakthrough therapy” designation for patients with mCRPC with *BRCA1/BRCA2* mutations previously treated with a taxane or AR-targeted therapy; this designation was based on the 41% overall response rate in comparison to 9% in non-*BRCA1/BRCA2* positive patients (GALAHAD study) [64]. Of note, TRITON2 and GALAHAD did not emphasize germline mutations.

The relevance of MMR mutations in mCRPC has also been evaluated following the FDA’s recent histology-agnostic approval of pembrolizumab for high microsatellite instability (MSI-high) and treatment refractory solid tumors [65]. Abida et al. reported that 7 of 32 patients with MSI-H or dMMR prostate cancer had a potential pathogenic germline mutation in MMR-associated genes, including *MSH2* (*n* = 5), *MSH6* (*n* = 1), and *PMS2* (*n* = 1). Of note, radiologic responses were reported in 4 of 11 patients with MSI-H/dMMR CRPC following treatment with PD1/PDL1 therapy (36.3%) [66]. Similarly, a recent study assessing pembrolizumab efficacy in patients with MSI-H mCRPC found 4 of 9 patients (44%) experienced a decline in PSA of 50%; interestingly, 2 of the 4 patients with response had pathogenic alterations in *BRCA1/BRCA2* [67]. The NCCN does not currently recommend germline detection of MMR mutations for clinical decision making [47].

### Companion Diagnostics

For the assessment of molecular indications for olaparib and rucaparib in mCRPC, several companion diagnostics are currently cleared by the FDA for use in patients with mCRPC [68]. For the detection of specifically germline *BRCA1/BRCA2* mutations in whole blood, the BRACAnalysis CDx platform provided by Myriad Genetic Laboratories can be used to select patients for treatment with olaparib [69]. More broad assessments of potentially clinically relevant markers are offered by Foundation Medicine. Designed to detect the relevant germline and/or somatic biomarkers in biopsy tissue validated in the PROfound study, the FoundationOne CDx platform can be used to select patients for olaparib treatment [70]. The FoundationOne Liquid CDx platform, which uses a plasma sample instead of biopsy, can be used for the detection of *BRCA1/BRCA2* mutations for treating patients with either olaparib or rucaparib, while also detecting *ATM* alterations relevant for olaparib therapy [71]. These companion diagnostics are summarized in Table 3.

## 5. Genetic Counseling

Genetic counseling (GC) is an important aspect of patient care because it enables patients to fully understand the nature of their disease and the implications that a molecular test can have on their lives and their families’ lives. In PCa specifically, GC has become important for patients who have relevant family histories and/or are eligible for biomarker-driven treatments [72]. Between 1 and 2% of patients with PCa present with a family predisposition and could benefit from optimized pharmacotherapeutic options [73].

**Figure 1:**
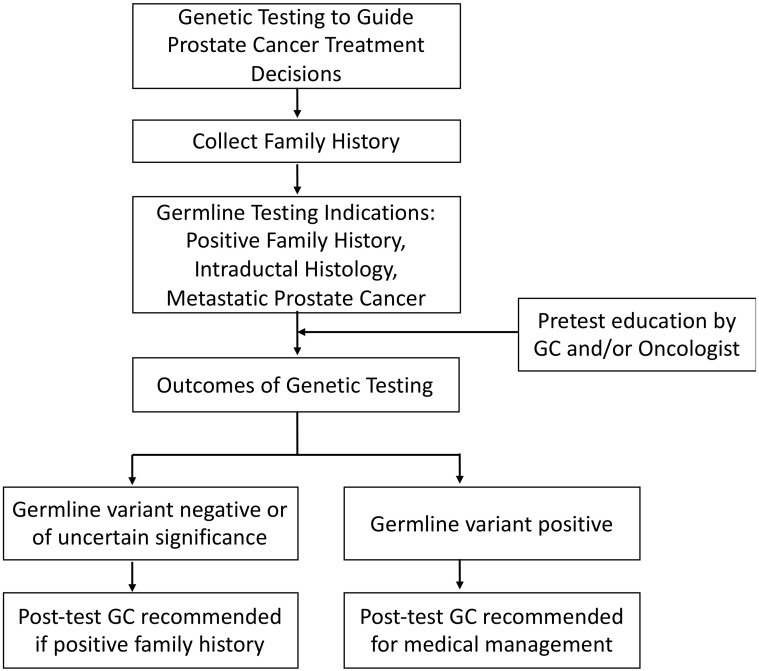
General framework for genetic testing to guide prostate cancer treatment decision. Refer to text for detailed description. GC = genetic counseling.

A general framework for genetic testing is provided in Figure 1. Two models of GC exist to assist patients, each focusing on different patient specific factors. The traditional model uses an upfront referral for GC by a nongenetic provider, who identifies the need for GC and can refer the patient to a genetic specialist for pre-test counseling, genetic test ordering and testing, and post-test disclosures. This model ensures the genetic counselor manages each stage of the process with their expertise. In contrast to the traditional model, the collaborative, hybrid model better integrates nongenetic providers into the process; under this model, the nongenetic provider recognizes a patient who may be in need of GC, collects pre-test informed consent from the patient, and then orders the genetic tests. The non-genetic provider then reviews the genetic test results with the patient and determines appropriate management strategies, including providing a referral to a genetic specialist to further discuss the findings and recommendations regarding family history as needed. If the results are pathogenic, referral to a genetic specialist is provided to further assess the positive results and family history-based recommendations upon a comprehensive assessment of the patient’s family history. In cases where the results reflect a variant of uncertain significance or a negative result, the primary provider reviews the results with the patient and makes recommendations based on family history [48].

The NCCN Prostate Cancer guidelines currently recommends the use of GC, especially a pre-test, when there is a positive family history. A post-test GC is recommended when a germline PV has been identified in a patient. In cases where there is not a pathogenic variant or a variant of unknown significance is found, but there is a positive family history, GC is recommended to determine if family-based studies should be completed. It is explicitly stated that if a patient is positive for *BRCA1, BRCA2, ATM, PALB2*, or *CHEK2* gene mutations, it is imperative to refer the patient to a GC for confirmatory testing [47].

Cascade testing, or testing of relatives for pathogenic gene mutations, is important to inform family members and ease the worry and anxiety associated with positive family history. It allows the opportunity to determine the risk and predisposition for of malignancy, and it will enable providers and genetic counselors to begin recommending family history-based care earlier on in the process [47,74].

## 6. Challenges and Barriers to Prostate Cancer Genetic Testing

Based on results of a 14-item questionnaire sent to academic oncologists at 43 different Prostate Cancer Clinical Trials Consortium locations, access to GC, insurance coverage, clinic workflow processes, space and time availability, as well as access to provider and patient education materials were cited as the main barriers for considering and obtaining germline testing for PCa patients [75]. As a result, there is a definite need for more genetic counselors and provider education on the importance and relevance of GC. In another study, 60% of providers stated they lacked the knowledge regarding genetic testing and genetics to provide adequate care and care coordination for their patients [76]. It is important to not only recognize these barriers but to also find solutions. With the increase in cancer treatments becoming more targeted at a molecular level, the utility of GCs is invaluable in assuring patients have a thorough understanding of their disease, treatment options, and familial impacts. In addition to the many members of the multidisciplinary healthcare team, incorporating GCs into outpatient oncology clinics and making them part of the clinic workflow for new visits and follow-ups needs to be a priority. Insurance companies will be late adapters of the including GC into the work-up of the patients they cover; however, with more convincing outcomes data, we believe this shift will occur. Lastly, direct patient discussions about the resources available for genetic testing and GC patient education needs to be better incorporated into patient visits.

## 7. Conclusions

The treatment and overall management of PCa is moving towards the implementation of precision medicine. Much progress has been made over the last several years in the treatment armamentarium that has resulted in the inclusion of recommendations regarding genetic testing and genetic counseling in the latest PCa guidelines. With PARP inhibitors and anti-PD1/PDL1 inhibitors included as treatment options based on genetic mutations, as well as the continuation of clinical trials and research finding new targets and patient-specific mutations, we see a paradigm shift in the drug management of PCa. Expanding access to gene-related care and therapy options as well as continuing to decrease disparities in PCa should be at the forefront of PCa research.

## Figures and Tables

**Table 1:** Prevalence of germline DDR variants in patients with prostate cancer reported in selected studies.

Prevalence of Germline Variants (%)					
Gene	Established Association with PCa Risk	Pritchard et al. [17]	Nicolosi et al. [35]	Robinson et al. [19]	* Chung et al. [36]
		Patients with mPC (*n* = 692)	Patients with PC (*n* = 3607)	Patients with mCRPC (*n* = 150)	Primary or Metastatic PC Tumor Samples (*n* = 3476)
*ATM*	yes	1.6	2.0	0.67	1.9
*BRCA1*	yes	0.9	1.3	0.33	0.35
*BRCA2*	yes	5.4	4.7	5.3	5.7
*BRIP1*	-	0.2	0.3	-	-
*CHEK2*	yes	1.9	2.9	-	1.4
*FANCA*	-	-	-	-	0.68
*NBN*	-	0.3	0.3	-	-
*PALB2*	-	0.4	0.7	-	-
*RAD51B*	-	0.3	-	-	-
*RAD51C*	-	0.14	0.2	-	-
*RAD51D*	-	0.4	0.2	-	-

mPC, metastatic prostate cancer; PC, prostate cancer; mCRPC, metastatic castration resistant prostate cancer * Analyzed from tumor tissue originating from either primary or metastatic sites. Percentages were calculated based on reported data.

**Table 2:** Prostate cancer testing panels.

Prostate Cancer Genetic Tests			
Test Name	Company	Genes Tested	Type of Sample
Prostate Cancer Panel [52]	Invitae	*ATM, BRCA1, BRCA2, CHEK2, EPCAM, HOXB13, MLH1, MSH2, MS6, NBN, PMS2, TP53* -Add-on panel (*ATR, BRIP1, FANCA, GEN1, PALB2, RAD51C, RAD51D)*	Whole Blood or Buccal Swab or Saliva
Prostate Cancer Comprehensive Panel [53]	Fulgent	*ATM, BRCA1, BRCA2, CHEK2, EPCAM, HOXB13, MLH1, MSH2, MSH6, NBN, PMS2, TP53*	Whole Blood or Buccal Swab or Saliva
Prostate Next [54]	Ambry Genetics	*ATM, BRCA1, BRCA2, CHEK2, EPCAM, HOXB13, MLH1, MSH2, MSH6, NBN, PALB2, PMS2, RAD51D, TP53*	Whole Blood
Hereditary Prostate Cancer Panel [55]	GeneDX	*ATM, BRCA1, BRCA2, BRIP1, CHEK2, EPCAM, HOXB13, MLH1, MSH2, MSH6, NBN, PALB2, PMS2, RAD51C, RAD51D, TP53*	Whole Blood or Buccal Swab
Prostate Cancer Panel [56]	Prevention Genetics	*ATM, BRCA1, BRCA2, BRIP1, CHEK2, EPCAM, HOXB13, MLH1, MSH2, MSH6, NBN, PALB2, PMS2, RAD51C, RAD51D, TP53*	Whole Blood
myRisk [57]	Myriad Genetics	*APC, ATM, AXIN2, BARD1, BMPR1A, BRCA1, BRCA2, BRIP1, CDH1, CDK4, CDKN2A, CHEK2, EPCAM, GREM1, HOXB13 GALNT12, MLH1, MSH2, MSH3, MSH6, MUTYH, NBN, NTHL1, PALB2, PMS2, POLE, POLD1, PTEN, RAD51C, RAD51D, RNF43, RPS20, SMAD4, STK11, TP53*	Whole Blood or Saliva
Hereditary Cancer Panel [58]	Color	*APC, ATM, BAP1, BARD1, BMPR1A, BRCA1, BRCA2, BRIP1, CDH1, CDK4, CDKN2A, CHEK2, EPCAM, GREM1, MITF, MLH1, MSH2, MSH6, MUTYH, NBN, PALB2, PMS2, POLE, POLD1, PTEN, RAD51C, RAD51D, SMAD4, STK11, TP53*	Saliva

**Table 3:** Companion diagnostics for prostate cancer.

Companion Diagnostics			
Test Name	Company	Genes Tested	Type of Sample
* BRACAnalysis CDx [69]	Myriad Genetics	*BRCA1, BRCA2*	Whole Blood
* FoundationOne CDx [70]	Foundation Medicine	*BRCA1, BRCA2, ATM, BARD1, BRIP1, CDK12, CHEK1, CHEK2, FANCL, PALB2, RAD51B, RAD51C, RAD51D* and *RAD54L*	Biopsy #
** FoundationOne Liquid CDx [71]	Foundation Medicine	*BRCA1, BRCA2, ATM*	Whole blood

* FDA cleared companion diagnostic for olaparib—genes listed represent those relevant for the indication for PARP inhibitor therapy in patients with mCRPC [68]. ** FDA cleared companion diagnostic for Olaparib and rucaparib—genes listed represent those relevant for the indication for PARP inhibitor therapy in patients with mCRPC [68]. # Foundation Medicine detects both somatic and germline alterations but does not differentiate between the two on reports.
